# Immune checkpoint inhibitor-induced inflammatory arthritis: a qualitative study identifying unmet patient needs and care gaps

**DOI:** 10.1186/s41927-020-00133-8

**Published:** 2020-08-01

**Authors:** Laura C. Cappelli, Suzanne M. Grieb, Ami A. Shah, Clifton O. Bingham, Ana-Maria Orbai

**Affiliations:** 1 Division of Rheumatology, Johns Hopkins School of Medicine, 5501 Hopkins Bayview Circle, Suite 1.B.1, Baltimore, USA; 2 Department of Pediatrics, Johns Hopkins School of Medicine, Baltimore, USA

**Keywords:** Immunotherapy, Cancer, Inflammatory arthritis, Quality of life, Social support

## Abstract

**Background:**

Patients treated for cancer with immune checkpoint inhibitors (ICI) may develop autoimmune adverse events, including ICI-induced inflammatory arthritis (IA). ICI-induced IA treatment requires balancing immune activation to fight cancer and immune modulation to control autoimmunity. Our objective was to learn how patients experience ICI-induced IA and potentially conflicting treatment decisions.

**Methods:**

Semi-structured interviews were conducted with participants with rheumatologist-diagnosed ICI-induced IA recruited from a longitudinal cohort. The interview guide probed the experience of diagnosis and treatment, symptoms and impact of ICI-induced IA, coping mechanisms, and treatment decision-making. Two researchers used an iterative coding process to identify themes through inductive thematic analysis and consensus. An overarching conceptual framework was derived from the qualitative analysis to identify care gaps perceived by patients, and inform future research.

**Results:**

Fourteen patients with ICI-induced IA participated in semi-structured interviews. Five overarching themes were identified: an awareness gap leading to delay in diagnosis of IA, descriptors of ICI-induced IA and relationship to other adverse events, emotional and quality-of-life impact of IA, fear and decision-making, and contextual factors including social support.

**Conclusions:**

As reported by patients, ICI-induced IA had a significant functional and emotional impact, even as compared to cancer and other ICI-induced side effects. Increasing awareness and integrated care of ICI-induced IA, and increasing social support are key targets for improving patient care. Additionally, more data on cancer outcomes in patients requiring immunomodulation for ICI-induced IA would help address fear and uncertainty for patients, and better support them through therapeutic decisions.

## Background

Inflammatory arthritis (IA) due to immune checkpoint inhibitor (ICI) therapy for cancer is a novel disease entity and a powerful example of conflict between therapeutic benefit and side effects. ICIs, which block negative costimulatory molecules and their ligands leading to unchecked T cell activation [1] targeting tumors, have provided hope for improved prognosis in many advanced cancers. ICIs also cause inflammation in non-tumor tissue [2] and immune related adverse events, including inflammatory arthritis (IA) which is the immune-related adverse event most likely to be seen by rheumatologists [3–10].

For patients with cancer, side effects from chemotherapy, radiation and surgery can affect quality of life after treatment has ended [11, 12]. ICI therapy has been FDA-approved for less than a decade, and its impact on quality of life is just starting to be defined, especially in long term survivors [13]. IA has a variable temporal relationship to ICI therapy but can persist months to years after ICI cessation [14]. Pathogenesis, epidemiology, and clinical features of ICI-induced IA are incompletely defined, and the experience of patients has yet to be explored. Importantly, there is a unique context for patients with ICI-induced IA, as they are concomitantly being treated for advanced stage cancer. Though short term treatment with immunomodulation did not impact patient outcomes in melanoma treated with ipilimumab or nivolumab [15, 16], the theoretical concern of abrogating tumor response through immunomodulation for IA remains. A recent study showed that patients with non-small cell lung cancer had worse tumor response when they were treated with prednisone doses above 10 mg at the start of ICI therapy exemplifying potential negative effects of corticosteroids [17].

Our study explored the patients’ experience of ICI-induced IA in context of their cancer treatment and their approach to conflicting decisions. We propose a conceptual framework on which future research and interventions can be built.

## Methods

English-speaking adults (age 18 or older) with rheumatologist-confirmed ICI-induced IA were recruited from an ongoing single-center longitudinal observational study of rheumatic ICI-induced events for in-depth semi-structured interviews. All patients were treated with ICI agents (targeting CTLA-4, PD-1, and/or PD-L1). The study was approved by the Johns Hopkins Institutional Review Board (00066663, 00123172). The author team created an interview guide at the outset of the project to probe symptoms, impact, coping, awareness of ICI side effects, and decision-making. The guide was expanded during the study to explore in-depth topics discussed by participants. Specifically, additional questions about treatment decision making, understanding of friends and family about their illness, knowledge of ICI-induced IA as a side effect, and whether they had engaged with other immunotherapy patients to discuss their illness were added.

Semi-structured interviews were conducted in-person at an academic medical institution between February 2017 and November 2018, by a medical anthropologist (SG) and a rheumatologist with qualitative research expertise (AO) who were not involved in patient care and had not previously met the participants. Interviewers were selected for their experience in performing interviews for qualitative research and had no preexisting assumptions about the research topic. Potential participants who had previously agreed to be contacted for research opportunities were approached at in person clinic appointments or over the phone; 14 patients participated in interviews and one patient who consented dropped out due to scheduling issues. Interviews took place in a conference room outside of the clinic, lasted approximately 1 hour, were audio-recorded with participant consent, and field notes were taken. No one was present besides the researcher and participant. Phone interviews were conducted with two participants who could not have an in-person study visit due to multiple medical appointments and worsening cancer. No repeat interviews were performed. The audio-recording of each interview was transcribed verbatim, and no transcripts were returned to participants. The text was analyzed using an iterative, inductive thematic analytic approach. An initial coding framework was developed by two authors (SG, LC) by analyzing two transcripts and creating a preliminary coding framework via open coding. The codes identified were discussed and reconciled by the two researchers. Both researchers then independently applied the codes to a third transcript to ensure agreement on code definitions and data interpretation. One author (LC) then independently analyzed the remaining transcripts in Atlas.ti software (Cleverbridge, Chicago, IL) and recorded data saturation to inform further sampling. Once all transcripts were analyzed, three researchers (LC, SG, AO) discussed the synthesized text and developed the final hierarchical theme structure. Participants provided feedback throughout the interviews, and the final few participants provided input on the conceptual framework.

## Results

### Demographics and clinical features of participants

There were 14 participants with mean (SD) age of 53.7 (12.1) years, all Caucasian, and seven (50%) were women. Times to onset and diagnosis of ICI-induced IA were 9.14 (6.41) and 15.79 (8.30) months, respectively. The average clinical disease activity index for IA ranged from remission to high disease activity (median: 11.75, IQR: 3–24). Ten of 14 participants had additional immune related adverse events. Melanoma was the most common underlying cancer, followed by hematologic malignancies and solid tumors (Table 1).

**Table 1:** Participant demographics, oncology history, and inflammatory arthritis features

Patient	Gender	Race	Tumor type	Time to IA onset	Pattern of joint involvement	CDAI	IA treatment	Other irAEs	Lag time to ICI-induced IA diagnosis
1	Female	Caucasian	Melanoma	12 months	Knees, ankles, fingers	12.5	Current: NSAIDs Past: prednisone, methotrexate, sulfasalazine	Colitis	3 months
2	Female	Caucasian	Melanoma	8 months	Knees, hips, then small joints of hands, Achilles tendon	24	Current: methotrexate, prednisone	Colitis, sicca, rash, hypophysitis	10 months
3	Female	Caucasian	Endometrial cancer	16 months	Wrist, shoulder, hands. Prominent tenosynovitis	26	Current: prednisone	Eosinophilic gastritis, thyroid disease, rash	3 months
4	Male	Caucasian	Hodgkin lymphoma	2 months	Reactive arthritis: knees, ankles	12	Current: prednisone	Colitis, urethritis, conjunctivitis	1 month
5	Male	Caucasian	Melanoma	2 months	Reactive arthritis: knees, ankles, wrists	0	Current: adalimumab Past: prednisone	Colitis, urethritis, conjunctivitis	1 month
6	Female	Caucasian	Mycosis fungoides	16 months	Knee, wrist, MCPs, PIPs	11.5	Current: prednisone	Sinusitis, sicca	5 months
7	Male	Caucasian	Melanoma	6 months	Shoulder, MCPs, wrist	2.3	Current: adalimumabPast: prednisone	Pneumonitis, hypophysitis	8 months
8	Female	Caucasian	Melanoma	12 months	Knees, MCPs, PIPs	9	Current: methotrexate, sulfasalazine Previous: infliximab, prednisone, etanercept	Colitis, thyroiditis	1 month
9	Male	Caucasian	Duodenal Cancer	3 months	PIPs, wrists, shoulders	3	Current: methotrexate, prednisone	None	14 months
10	Male	Caucasian	NSCLC	7 months	Trigger fingers, PIPs, shoulders, wrist, inflammatory back pain	11	Current: sulfasalazine, NSAIDs	None	24 months
11	Male	Caucasian	Melanoma	6 months	Achilles enthesitis, PIPs, MCPs, extensor tendon tenosynovitis	25.9	Current: methotrexate	None	15 months
12	Female	Caucasian	Neuroendocrine carcinoma	13 months	PIPS, MCPS, wrists, knees	41	Current: prednisone	Rash	7 months
13	Male	Caucasian	Esophageal cancer	2 months	PIPs, MCPs, knees	3	Past: prednisone Current: NSAIDs PRN	None	0.5 months
14	Female	Caucasian	Melanoma	23 months	PIPs, wrists, elbows, ankles, MTPs	23.5	Current: NSAIDs Past: prednisone, adalimumab	Vitiligo, hypothyroidism, hepatitis	0.5 months

### Thematic analysis

Participants conceptualized their experience in terms of their cancer diagnosis, undergoing treatment for cancer, developing ICI-induced IA symptoms and life impact, and treatment for ICI-induced IA. Overarching themes are summarized below.

#### Awareness gap and diagnostic delay

**Fig. 1:**
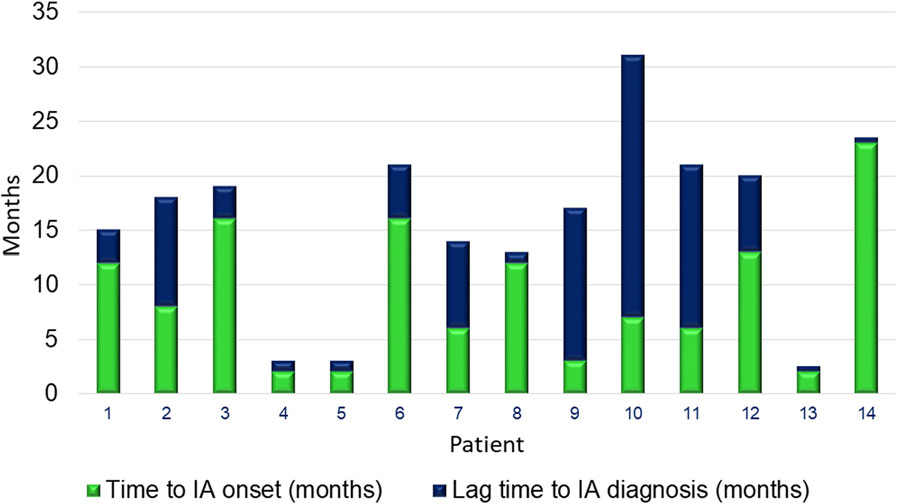
Time to IA symptom onset and IA diagnosis from initiation of ICI therapy. Time in months is represented on the Y-axis and participants are represented on the X-axis

A delay in diagnosis of ICI-induced IA was common (Fig. 1). Diagnostic delay would not necessarily be expected when side effects are consistent with the mechanism of action of a therapy, and patients are followed longitudinally within the medical system. Several participants spoke about symptoms spanning over months or going to many different healthcare providers before ultimately being diagnosed. The factors contributing to diagnostic delay included lack of information/misinformation, symptom attribution to family history or prior musculoskeletal injuries, the heterogeneous course of ICI-induced IA, and the fact that ICI-induced IA is still a rare entity and its recognition remains dependent on the level of expertise of healthcare providers.

The majority of participants described being unaware at the start of their ICI therapy that IA was a potential side effect. Other participants could not recall details of their discussions with healthcare providers, attributing this to being overwhelmed by their cancer diagnosis and prognosis. Without knowledge of the risk of ICI-induced IA, identifying other possible explanations for musculoskeletal pain was common.

“I thought I had regular arthritis [ … ] my family does have arthritis in its past so I figured some of that was from that” (Pt9, M)

Variability in the natural course and onset of ICI-induced IA was linked to under-recognition:

“I had forgotten about the side effects. It was after a year and a half. You think that you’re safe” (Pt6, W).

Some participants reported they perceived lack of knowledge about ICI-induced IA from the part of their healthcare providers, or that the level of expertise was too narrow for any one specialist to make the connection; while other participants described that their oncologist was able to recognize the ICI-induced IA immediately.

“[I] suffered a long time ‘til [I] found out, and went to at least a dozen different doctors trying to find the entity because [each doctor] was treating [considering] each as a separate entity” (Pt6, W).

“He knew right away what it was. He had another patient that had the same problem happen, so he knew right away that it was arthritis inflammation.” (Pt14, W).

#### Descriptors of ICI-induced IA and relationship to other adverse events

**Fig. 2:**
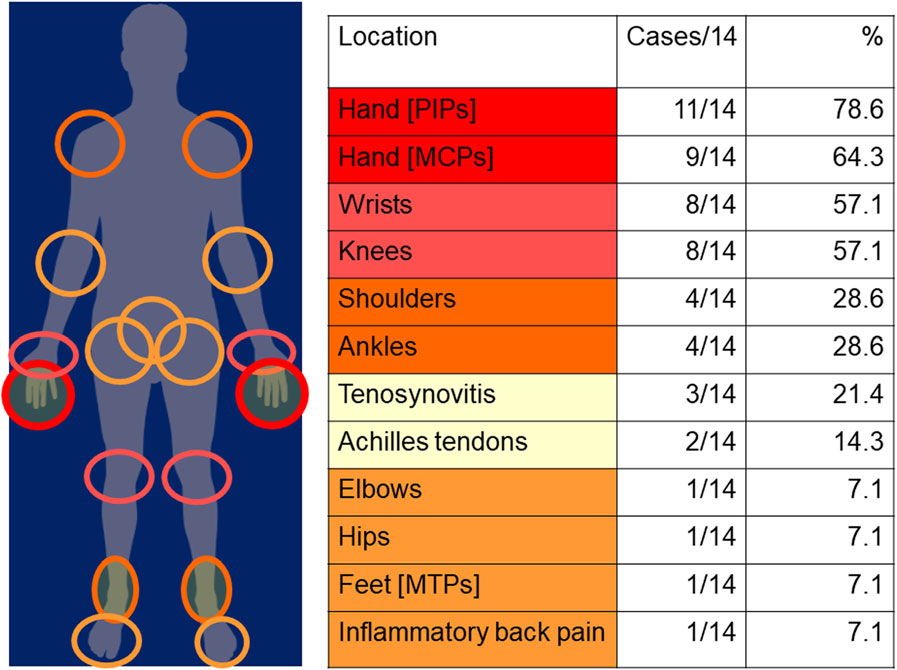
Patterns of musculoskeletal involvement in participants with ICI-induced inflammatory arthritis in the study

Descriptors of ICI-induced IA included the location of arthritis, quality of pain, associated symptoms, aggravating/alleviating factors (Table 2), and functional impact. The onset and course of ICI-induced IA varied, with some having an abrupt onset in days to weeks and others having a more gradual onset over months. Several patients reported an additive course where new joints continued to be affected. Patterns of joint involvement were variable (Fig. 2) and included as the most frequent manifestation hand PIP and MCP joint arthritis, followed by arthritis of the wrists and knees. Tenosynovitis/enthesitis were also described:

“Stage 4 melanoma: liver, lung and brain, they gave me 1 to 3 months. That takes the wind out of you, I tell you that. But getting to the arthritis part, the first thing that flared up was my tendons-- I guess the Achilles tendon-- so I could hardly walk. [ …] So, there came a point where I went out on disability, not because of the cancer itself, but because of the side effects from the cancer treatment. So, to say how [ICI-induced IA] affected my life, oh, my God!” (Pt11, M).

**Table 2:** Description of Arthritis symptoms

Category	Descriptors
Symptoms	Pain
	Joint Swelling
	Stiffness or “locking of limbs”
	Fatigue
	Weakness (e.g. in hands)
	Erythema of affected joint
Locations	Knees
	Fingers
	Wrists
	Ankles
	Feet
	Hips
	Shoulders
	Elbows
	“virtually every joint”
	“places where I had old injuries”
Onset/Course of arthritis	Very acute
	Gradual worsening over months, even after treatment ended
	Progressive
	Migratory
Descriptors of Pain	Intense
	Horrible
	Inflamed
Aggravating factors	Worse in dominant hand
	Morning (worse for stiffness)
	Fatigue worse as day goes on
	Exercise
	Cold weather
Alleviating factors	Better when distracted
	Ice
	`Medicine (NSAIDs, prednisone, DMARDs)
	Hot shower

A unique feature for this group of patients was that many experienced multiple ICI-induced autoimmune side effects and all noted that IA was one of their worst, because of its persistence, unpredictability/flares, and impact on function and health-related quality of life:

“[IA] continues to be a much bigger problem than those other side-effects [thyroiditis, colitis], as far as the level in which it affected my body, the length of time that I’m dealing with it and the effect it has on my overall quality of life, by leaps and bounds, a bigger deal than the thyroid or colitis issues” (Pt8, W).

“The other [gastrointestinal side effects] come and go. The other ones can be treated. This one (IA) can be treated, but then it shows up somewhere else” (Pt10, M).

How the arthritis compared in impact to other side effects appeared to be unpredictable and worse with IA flares. Also, resolution of ICI-induced IA and other concomitant ICI-induced events was not simultaneous and IA was described to lag behind other autoimmune manifestations:

“[IA impact is] really hard to quantify, or put a gradation. But when the arthritis was bad, it was astoundingly bad, and when that happened, all the other side effects from chemo had pretty much dissipated, except for the swelling and the neuropathy” (Pt9, M).

“[IA and gastritis were] initially on par, but now, I think I got better from [gastritis][but] maybe it’s just going to take longer [for arthritis]” (Pt3, W).

#### Emotional and quality of life impact in ICI-induced IA

Participants described IA affected them socially and emotionally, while the experience of cancer and cancer treatment had its own impact. Positive feelings, as well as distress, were described as participants recounted their experiences. Some expressed little additional emotional impact from IA beyond cancer.

Other participants discussed their advanced cancer diagnosis as placing the arthritis in perspective and reported feelings of gratefulness and hope, even exuberance:

“I’m here. I’m lucky. That’s probably the biggest thing [ …] I’m still walking on this earth after four years” (Pt10, M).

“Because I’m alive! [ …] that’s what changes you [...] when I was there before, there was a fence here [ …], because no matter where we’re at, you are in the land of the living, I’m in the land of the walking dead. I have a timeline. I don’t know if I’m going to see, when those leaves fall off, am I going to get to see them next spring? Now, that’s been removed. I’m cancer-free. I’m this, that, and the other.” (Pt11, M).

Others, however, reported emotional distress related to developing ICI-induced IA after already enduring cancer and cancer treatment. This was frequently linked with decreased physical function and disability caused by the ICI-induced IA:

“[The arthritis] knocked me back a lot harder than honestly the cancer diagnosis initially, because I could not move. I was on the sofa or in bed for days without knowing why or if this was ever going to get any better. It was terrifying.” (Pt8, W).

“[The arthritis] affected me in a very strong way emotionally, because … I didn’t feel independent. I became very dependent.” (Pt6, W).

Several participants described personal growth and how finding support structures, whether in their family or friendships, increased their strength and maintained their psychological health:

“It’s the same thing when they told me that I was sick. It’s like, oh God, I still want to be around for my kids. I guess my willpower. [ …] My son, senior year, was terrible. He was very good in two sports so I missed the one sport completely. But it’s okay because I overcame this and I was able to see the rest of it. And I shouldn’t be crying because I’m okay. What are you going to do? You hit bumps in life. You’ve just got to overcome them.” (Pt12, W).

“If I wake up and I’m hurting and I’m swollen and I don’t feel right she’s like, <You’re good and we’ll stay home and we’ll try to put off doing what we have to do.> I have a lot of good support.” (Pt14, W).

#### Fear and decision making

Once experiencing ICI-induced IA, participants described varying amounts of involvement in the decision to stop or continue the ICI therapy for their cancer. For participants in clinical trials, depending on the protocol, there was a requirement to come off therapy for serious adverse events, or they were given the choice to continue or stop. Fear of uncertainty, often rooted in the assumptions that their cancer would come back, was discussed. Fear was mentioned as influencing three key decision-making points for patients: 1) continuation of ICI therapy for cancer, 2) reporting of symptoms of ICI-induced IA to their physicians, and 3) beginning or continuing immunomodulation therapy for ICI-induced IA.

“I don’t know, I might have decided to continue because I was super scared of being off treatment. I was just really afraid of the melanoma coming back.” (Pt8, W).

“I was only a few months into [the immunotherapy], but the change [in the cancer] that they had seen was so drastic and so fast. And I stayed on the immunotherapy drug … and at that time, the cancer could have been gone, but I didn’t even want to stop then …. I thought that if I told them that I was in pain, they would stop giving me the immunotherapy, and I wasn’t going to have that.” (Pt11, M).

Participants who had complete tumor remission described an understanding that there was limited benefit in receiving more ICI therapy. A participant described a shift in her feelings to be more concerned with side effects after a good tumor response:

“I didn’t want to take the risk, since I was very close to the end, of going back on [ICI therapy] and possibly experiencing more of the side-effects.” (Pt6, W).

Some participants who had not had a positive tumor response and required additional cancer therapies, were more cautious in cancer treatment decisions.

“[I’m] somewhat anxious about starting yet another medicine and it’s like I don’t need another disease. I have enough going on. So there’s a little trepidation about starting another one or I really want to understand the side effects and I keep hoping that this will abate.” (Pt3, W).

The decision to use immunomodulation for IA treatment required weighing fear of their cancer returning versus ICI-induced IA impact. Participants reported reading and having been counseled that drugs like disease modifying anti-rheumatic drugs/biologic disease modifying anti-rheumatic drugs (DMARDs/bDMARDs) could lead to increased cancer and infection risk. Nonetheless, some participants reasoned DMARD/bDMARD may be the preferred choice when ICI-induced IA is active and impactful and should be available. One participant reported significant IA relief with bDMARD which was meaningful to her.

“I think it’s important for rheumatologists to understand that when they’re dealing with someone who’s had a cancer diagnosis that fear is a really big factor when talking about [immunomodulation] treatment.” (Pt8, W).

“Just like with any meds, you’re going to have to hope you don’t get that side effect. <laughs> I mean, as many meds as I’ve been on, you hope and pray that you don’t get that side effect, and take the chance, and hopefully the medicine works and the side effects aren’t as bad as they say they’re going to be. And it wasn’t too bad compared to the outcome of the effect [the bDMARD] gave me. It took all the inflammation away so it was amazing. It took a couple months for it to work but after it started working, it was amazing.” (Pt14, W).

#### Contextual factors including social support

Participants described experiencing that symptoms and impact of ICI-induced IA were frequently underestimated by friends and family in the presence of a concomitant cancer diagnosis. Several possible reasons were discussed including that immunotherapy was in general better tolerated than other cancer treatments; misattribution of arthritis to other etiologies than immunotherapy; and pre-conceived notions about the importance of improving survival from cancer as the ultimate goal of care. These are described below:

“I think that it’s really easy for someone to understand that if you have cancer you may not be able to do certain things or function in the same ways, but [arthritis] might not be as understood, and so I may have a colleague or two who doesn’t quite understand why I’m not as physically agile.” (Pt8, W).

“Immunotherapy doesn’t make you look ill. People think, <Well, you’re fine; you’re great, and you’re done with treatment> [ …] and I couldn’t do anything.” (Pt2, W).

Other participants suggested that arthritis was common and not viewed as an impactful health problem. One participant shared that friends downplayed the significance of IA because of a misattribution to normal aging:

“Well, nobody is afraid to question that [the arthritis] has something to do with cancer. But on the other side of the coin they’re saying, ‘You’re getting old.’ You know, that’s pretty much their basic canned answer.” (Pt10, M).

An additional explanation was that friends and family assumed an exclusive focus on death from cancer so that any other outcome from treatment seemed positive in comparison. Regardless of how participants understood this difference in social support, they were emotionally impacted by lack of support:

“As far as people’s reaction to the arthritis, it bummed me out [ …] their perspective is obviously different from mine [ …] for minimizing my having arthritis.” (Pt9, M).

“I think cancer gives people a free pass with a lot of things. But there’s so many other illnesses that are chronic and disabling and really limit people and I think this has been an eye-opener for me.” (Pt3, W).

### Conceptual framework of the experience of ICI-induced IA

**Fig. 3:**
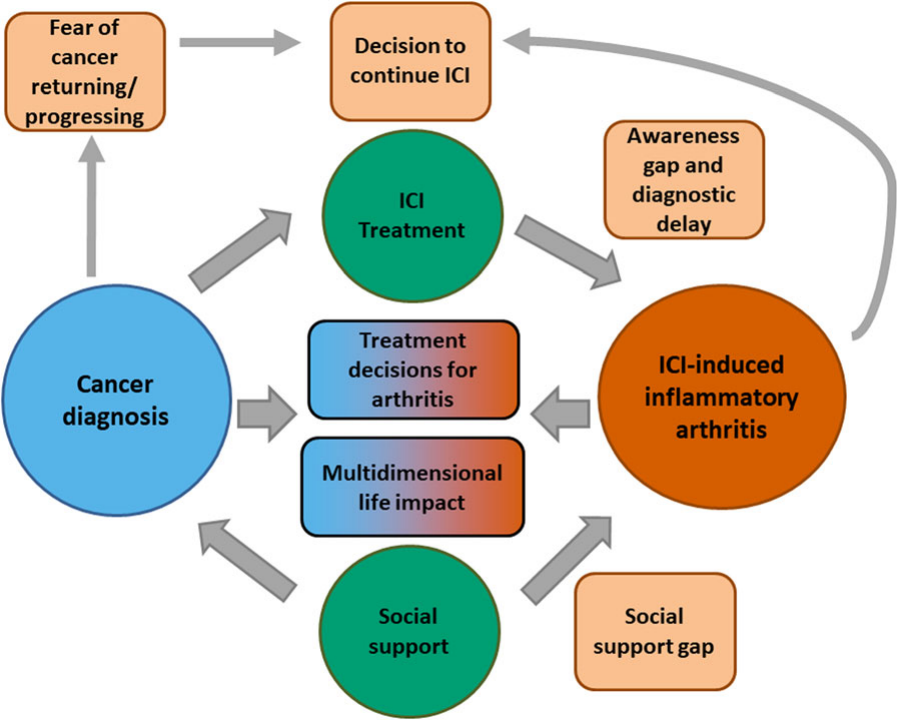
Conceptual framework for patient experiences with immune checkpoint inhibitor-induced inflammatory arthritis

As depicted in the Fig. 3, ICI treatment can lead to IA, but diagnosis is frequently delayed by an awareness/knowledge gap affecting both patients and providers. People with cancer treated with ICIs who develop IA, and their medical doctors, are faced with a decision dilemma where they must consider not only their cancer diagnosis but also symptoms and impacts of ICI-induced IA when deciding whether to continue ICIs and how to treat emerging IA. Fear of cancer returning or progressing plays an important role in patients’ willingness to stop ICI therapy. Patients face multidimensional life impact from their two diagnoses, cancer and ICI-induced IA, but may experience less social support for their IA.

Table 3 provides additional illustrative quotes.

**Table 3:** Quotations from patients with ICI-induced IA representing five themes

Theme	Illustrative quotes
1. Awareness gap and diagnostic delay	*It took me by such surprise, I think. You get so many handouts and literature on immunotherapy […*] *but they didn’t have anything about [arthritis].* Pt 7 (F) *In fact, one of the selling points, so to speak, was that, “Oh, immunotherapy doesn’t have any side effects.* Pt 9 (M) *[The doctor] said, “Oh nobody else has [arthritis]-- I haven’t seen that on anybody else.”* Pt 12 (F)
2. Descriptors of ICI-induced IA and relationships to other adverse events	*My hands, I couldn’t bend my hands […*] *[they] were red and inflamed. I couldn’t open up doors, turn a wheel, open a car door. I couldn’t open any kind of bottles. I had trouble picking up anything at all. I never had anything in my life and I still don’t. I have this cancer and they treated me with it. And removed it. And then I was good. I was on [ICI therapy] just about 3 years and then I had to stop it because, oh my God, I went to see my regular doctor so that I could get slips so that I could go for physical therapy because I was losing the use of my hands completely.* Pt 12 (W) *You kind of think of the arthritis as-- if your elbow and hand doesn’t work you’re like, oh my God, is that going to be like that the rest of my life? Whereas I’m not worried about having diarrhea the rest of my life. You can be treated or whatever.* Pt 10 (M) *Well, the fatigue and the arthritis are, you know, always present in the background. The colitis, I haven’t had a problem with.* Pt 5 (F) *My hands were acting up. They probably got severe this year, early in the year […*] *and then it kept coming like a lion at me. I came down here and then I started the prednisone and the hands started backing off, and then all of a sudden it went from my hands. […*] *That’s what it is. It was backing away and then it’s fierce.* Pt 12 (W)
3. Emotional and quality of life impact in ICI-induced IA	*To be told that you have arthritis was odd. [I] wasn’t upset. And I hope that I have a long, a lot longer, time to live.* Pt 2 (W) *I was pretty devastated because up until that point where the knee pain started, I had made a really big recovery from the beginning of the whole process of being diagnosed and the big surgeries that I had right away*. Pt 8 (F) *It was just very limiting. Was very frustrating. Because I felt like I was a hundred. I fought for two and a half years to beat cancer and then I can’t do anything.* Pt 2 (F) *I can live with it until the day that it completely debilitates me for some reason. I’m still here. So that’s all that matters.* Pt 10 (M) *I found each of those hobbies more difficult to do. I couldn’t hold certain tools, and I’d be dropping screws, and it led to a lot of amount of frustration. I’d have to stop, because I didn’t want to get all frustrated and get all in a sour mood.* (Pt 9, M)
4. Fear and decision making	*[The doctor] was just trying a lot of different medications and combinations of medications [for the arthritis], and also kind of having to deal with the unknown of how this would affect my melanoma, so [she was] having to be really careful about that and dealing with my fears of how that’s going to affect my melanoma.* Pt 8 (F) *I would be hard pressed to say that I would have continued as long as I did in the trial if I had known [about the side effects]. The other option, of course, is to be dead, but you know at some point it becomes a toss up.* Pt 1 (F) *My hope with the immunotherapy was […*] *that if any little seeds of cancer had spread elsewhere in my body, or remained in my body, that the immunotherapy would knock those out and I’d get a clean start. And at this point if I would have had to reverse the effect of the immunotherapy, I would consider it*. Pt 13 (M) *And [my oncologist] doesn’t say anything directly [only] makes comments < If you choose to suppress your immune system like that > […*] *you’d be sitting there thinking, <He doesn’t think I should be doing that>. […*] *I don’t know what I’m going to do. […*] *What I don’t want to do is continue down a path where I’m taking a drug [DMARD] that can give me cancer that no one can tell me if it’s going to work or not.* Pt 11 (M)
5. Contextual factors including social support	*Other people would say, “Well, compared to being dead from cancer, having arthritis, this isn’t that bad.” But in retrospect, it’s easy to say, when you don’t have it.* Pt 9 (M) *During the immunotherapy phase I was getting my strength back and […*] *my family especially were very excited […*], *and plus them being so far away they weren’t really seeing me. I’d have a friend take a picture of me but only when I felt like I was looking better < laughs > and so they were all very optimistic about it, concerned but optimistic at that point, whereas before they were very concerned. And during the arthritis part, I don’t even think they were aware of that*. Pt 13 (M)

## Discussion

To our knowledge, this qualitative study is the first to explore the experiences of participants confronted with a new rheumatologic entity, ICI-induced IA. Participants who had delay in diagnosis attributed this to lack of awareness of IA as a side effect of immunotherapy. The arthritis was a significantly morbid event for most, even considered in the context of concomitant advanced stage cancer. Diagnosis and treatment decision-making were complicated and sometimes difficult to navigate. Fear about cancer returning influenced decision-making and was potentiated by lack of data/unanswered questions that would otherwise have allowed these fears to be better analyzed and addressed. Finally, patients with ICI-induced IA perceived less social and other support in managing and coping with their arthritis than they had received for their cancer.

Previous qualitative studies have explored the experiences of cancer survivors, a similar group to most patients in this study who had a positive tumor response to ICI therapy. Among cancer survivors, quality of life is influenced by numerous factors, several of which may be impacted by the co-occurrence of ICI-induced IA. Fear of cancer recurrence, for example, can negatively affect quality of life by itself [18]. This fear is compounded by lack of data on combining ICI therapy with immunomodulation and cannot be effectively addressed by healthcare providers. Research in melanoma has shown that even after cancer treatment is completed, ongoing physical problems and uncertainty about cancer recurrence continue to create emotional distress for patients [19, 20]. These factors are particularly important in patients with ICI-induced IA since the arthritis can cause persistent physical problems, and concerns about cancer recurrence with immunomodulation or stopping ICI therapy cause difficulty in decision making. Physical activity has been shown to positively impact quality of life for cancer survivors [21–23], so the inability to be as physically active as desired in those who develop ICI-induced IA may be a key barrier to improved quality of life.

Limitations of the study include the small sample size and sample enrichment for responders to ICI therapy. This survival bias is inherent to studying long-term effects of cancer treatment. However, the experience of long-term survivors with persistent IA are likely the most relevant for rheumatologists who will become involved in their longitudinal care. Participants were Caucasian and treated at an academic medical center, which may limit the generalizability to other populations. Strengths of our study include sampling within a longitudinal cohort with detailed phenotypic data on both cancer and ICI-induced IA.

The study lays out several needs from the patient perspective that include educational, social and decisional support. In order to address the fear of immunomodulation that exists currently more research is needed on optimal duration of immunotherapy for efficacy, and on the impact of immunomodulation for ICI-induced IA on tumor response and patient survival. Additionally, patients with ICI-induced IA may benefit from early rheumatologic care that would recognize, measure, and treat IA longitudinally.

## Conclusions

In this study of ICI-induced inflammatory arthritis, participants attributed delay in diagnosis to under-recognition of IA as a side effect of ICIs, and to variability in its presentation. ICI-induced IA had significant functional, social, and emotional impact as compared to cancer and other side effects. Participants perceived less social and decisional support for their ICI-induced IA than for their cancer diagnosis and treatment. In order to improve the medical care of patients with ICI-induced inflammatory arthritis, we need to implement integrated clinics, build educational and support structures for patients and their families, and conduct therapeutic studies to improve the evidence base.

## Data Availability

The data are available upon reasonable request.

## Abbreviations

ICI: Immune checkpoint inhibitor
IA: Inflammatory arthritis
W: Woman
M: Man
DMARD: Disease modifying anti-rheumatic drugs
